# Anhydrobiosis-Associated Nuclear DNA Damage and Repair in the Sleeping Chironomid: Linkage with Radioresistance

**DOI:** 10.1371/journal.pone.0014008

**Published:** 2010-11-16

**Authors:** Oleg Gusev, Yuichi Nakahara, Veronica Vanyagina, Ludmila Malutina, Richard Cornette, Tetsuya Sakashita, Nobuyuki Hamada, Takahiro Kikawada, Yasuhiko Kobayashi, Takashi Okuda

**Affiliations:** 1 Anhydrobiosis Research Unit, National Institute of Agrobiological Sciences, Tsukuba, Japan; 2 Department of Invertebrate Zoology, Kazan State University, Kazan, Russia; 3 Microbeam Radiation Biology Group, Japan Atomic Energy Agency, Takasaki, Japan; 4 Nuclear Technology Research Laboratory, Radiation Safety Research Center, Central Research Institute of Electric Power Industry (CRIEPI), Tokyo, Japan; The University of Hong Kong, Hong Kong

## Abstract

Anhydrobiotic chironomid larvae can withstand prolonged complete desiccation as well as other external stresses including ionizing radiation. To understand the cross-tolerance mechanism, we have analyzed the structural changes in the nuclear DNA using transmission electron microscopy and DNA comet assays in relation to anhydrobiosis and radiation. We found that dehydration causes alterations in chromatin structure and a severe fragmentation of nuclear DNA in the cells of the larvae despite successful anhydrobiosis. Furthermore, while the larvae had restored physiological activity within an hour following rehydration, nuclear DNA restoration typically took 72 to 96 h. The DNA fragmentation level and the recovery of DNA integrity in the rehydrated larvae after anhydrobiosis were similar to those of hydrated larvae irradiated with 70 Gy of high-linear energy transfer (LET) ions (^4^He). In contrast, low-LET radiation (gamma-rays) of the same dose caused less initial damage to the larvae, and DNA was completely repaired within within 24 h. The expression of genes encoding the DNA repair enzymes occurred upon entering anhydrobiosis and exposure to high- and low-LET radiations, indicative of DNA damage that includes double-strand breaks and their subsequent repair. The expression of antioxidant enzymes-coding genes was also elevated in the anhydrobiotic and the gamma-ray-irradiated larvae that probably functions to reduce the negative effect of reactive oxygen species upon exposure to these stresses. Indeed the mature antioxidant proteins accumulated in the dry larvae and the total activity of antioxidants increased by a 3–4 fold in association with anhydrobiosis. We conclude that one of the factors explaining the relationship between radioresistance and the ability to undergo anhydrobiosis in the sleeping chironomid could be an adaptation to desiccation-inflicted nuclear DNA damage. There were also similarities in the molecular response of the larvae to damage caused by desiccation and ionizing radiation.

## Introduction

Extreme environments force organisms to develop or adopt effective mechanisms of cellular and molecular protection. Anhydrobiosis, the ability of organisms to survive in the dry state, is one of the most advanced strategies among hypometabolic states [1], [2]. While the cells of other organisms subjected to dehydration exhibit massive damage to their organelles and membranes, anhydrobiotic organisms can effectively counteract the negative effects of water deprivation [3], [4], [5]. Although the molecular mechanisms underpinning anhydrobiosis are not yet completely understood, it is generally accepted that they involve two broad functions: effective preservation of cells and biomolecules under dry conditions; and recovery and alleviation of the negative effects, both direct and indirect, of water loss on biomolecules upon post dry-state rehydration [3], [6].

In addition, being anhydrobiotic confers cross-tolerance to various other extreme environmental stressors, including different types of radiation [7], [8], [9], [10], [11], [12], [13]. It has long been recognized that this is possibly due to the protective mechanisms associated with anhydrobiosis, including the physical protection (i.e. free radical partial scavenging and radiation shielding) of cells by sugars, LEA proteins and other protectants against direct irradiation or its side effects [7], [14], [15], [16], [17], [18]. Indeed, at least for microorganisms and cultured cells, it has been shown that coating with sugars, such as trehalose, increases survival after long-term exposure to UV and ionizing irradiation [19], [20], [21].

The enhanced protection and repair of DNA might also be responsible for the cross-tolerance to ionizing radiation [2], [7], [10], [13], [22], [23]. In plant seeds and desiccation-resistant bacteria, repair of fragmented DNA is an indispensable step in revival after anhydrobiosis [24], [25], [26]. In multicellular animals, the effect of anhydrobiosis on DNA is controversial. While prolonged dehydration affects the nucleic acids of anhydrobionts and plant seeds in a duration-dependent manner, anhydrobiosis does not cause any serious DNA damage in the anhydrobiotic nematodes, crustaceans and tardigrades with the exception of bdelloid rotifers in which the DNA fragmentation effect of desiccation is suggested to occur [8], [22], [27], [28], [29], [30].

The sleeping chironomid *Polypedilum vanderplanki*, which inhabit the semi-arid area in Africa, is the only insect with the ability to resist almost complete dehydration during its larval stage and to reversibly revive within an hour of re-hydration [31]. Since the artificial rearing method for this species was established [31], a significant progress has been made to understand its structural protective mechanisms during anhydrobiosis at cellular and molecular levels. During dehydration, trehalose and LEA proteins increase in quantity [31], [32], [33], replacing the water in cells and forming glasses to preserve the cell structure in the dry state [34]. Concurrently, the expression of genes encoding heat shock proteins (i.e., chaperones) is increased, resulting in the protection of other proteins from denaturation caused by dehydration [35].

In a series of irradiation studies [7], [16], [36], it has been demonstrated that dried larvae show higher tolerance to both high-LET (directly causing DNA breaks) and low-LET (causing increase of reactive oxygen species (ROS) in the irradiated tissues) irradiation, measured by short-term survival compared with physiologically active larvae. Furthermore, this enhanced radiation tolerance is observed in both desiccating larvae, and larvae immediately after rehydration [16], suggesting that radiotolerance mechanisms are in place during both the induction and the recovery phase of anhydrobiosis.

The aim of the present study is to evaluate and compare the effects of anhydrobiosis, gamma-ray and heavy-ion irradiation on the nuclear DNA and the gene expression of the larvae of *P. vanderplanki*. We demonstrate that larval DNA becomes fragmented both upon anhydrobiosis and irradiation, which is later repaired through rehydration or recovery from irradiation. Thus, the DNA repair ability associated with anhydrobiotic potential seems to correlate with radiotolerance of the chironomid larvae. In addition, analyses of gene expression and antioxidant activity suggest the importance of ROS removal and DNA repair systems to protect biomolecules from damages associated with water loss and gamma-rays.

## Results

### DNA fragmentation caused by irradiation and anhydrobiosis

**Figure 1:**
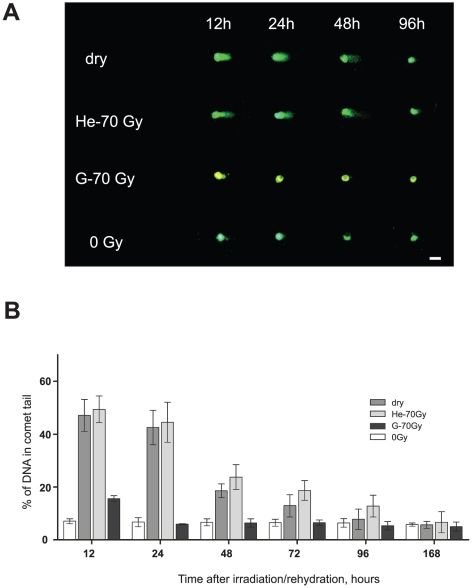
Time course of DNA repair in the fat body cells of hydrated larvae after ^4^He^+^ ion and gamma-ray irradiation and larvae rehydrated after three months of anhydrobiosis. (**A**) Typical comet images of nuclear DNA from fat body cells of larvae over a 96 h time course of recovery after irradiation by gamma rays (G-70 Gy) and ^4^He^+^ ions (He-70 Gy) to hydrated larvae, respectively and anhydrobiosis (dry: dehydrated larvae). The line marked “0 Gy” corresponds to nuclear DNA from intact hydrated larvae. Bar = 5 µm. (**B**) Proportion of DNA in the comet tail in the fat body cells of larvae irradiated by gamma rays or^ 4^He^+^ ions, or following rehydration after anhydrobiosis. Error bars represent mean value ±95% CI.

DNA breaks in fat body cells have been visualized and quantified using a comet assay method up to 168 h after exposure of the larvae to 70 Gy from two types of radiation (gamma-rays and heavy ions) and also during the recovery of dry larvae after re-hydration (Fig. 1A). Although some cells from non-irradiated hydrated larvae exhibited detectable levels of DNA fragmentation, probably reflecting naturally occurring breakage during the cell cycle or effects of experimental procedures, the mean level of background DNA fragmentation (% of DNA in the tail of a comet) in a pool of these control cells never exceeded 5–7% (Fig. 1B).

Irradiated larvae showed significantly higher levels of DNA fragmentation ranging from 15% to 50% depending on the type of radiation used (Fig. 1A, B). Comparative analysis of DNA recovery kinetics shows that it took up to 168 h for the larvae irradiated with heavy ions to recover nuclear DNA integrity to the control baseline level, whereas DNA of gamma-ray irradiated larvae was repaired within 24 h (Fig. 1A, B).

Non-irradiated dried larvae after rehydration also contained cells with severely damaged DNA, with a level of fragmentation comparable to the larvae exposed to 70 Gy ^4^He ions. In the anhydrobiotic larvae, however, DNA damage decreased to the background level within 96 h after rehydration, much quicker than in ^4^He ion-irradiated samples. While entering into the anhydrobiotic state always caused DNA damage in the larvae, no further significant increase in the level of nuclear DNA damage was found, even when the dry larvae were kept at room temperature for 14 months.

In both ^4^He ion-irradiated and post-anhydrobiosis larvae, no significant recovery of DNA was observed within 24 h (Fig. 1B). Despite this prolonged and severe fragmentation of DNA however, no large scale cell death was observed in either group.

### Ultrastructure of cells of hydrated and dry larvae

**Figure 2:**
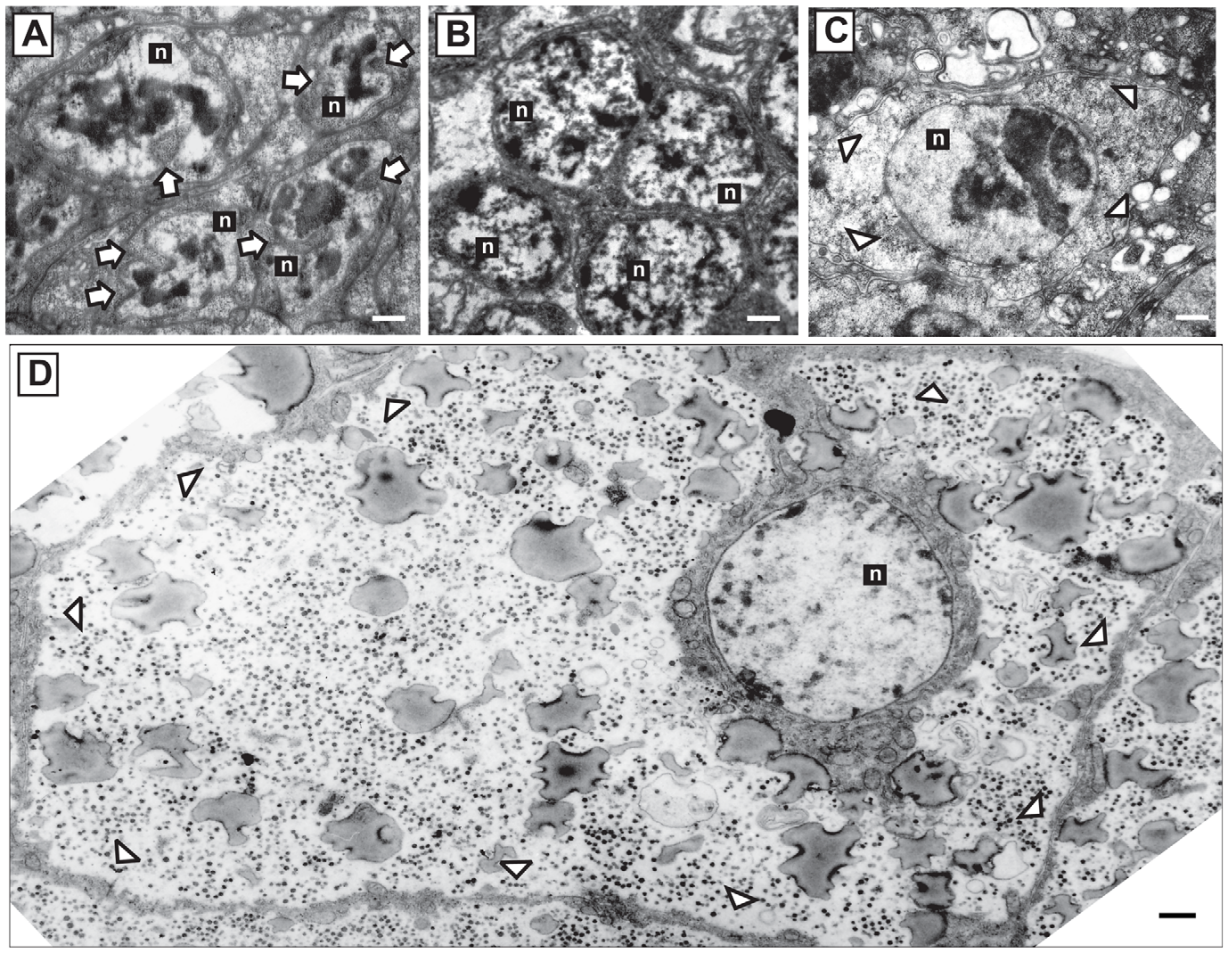
Ultrastructure of nuclei (n) of two cell types from dry and hydrated larvae. **A:** Cells of non-differentiated cell mass in a dry larva. **B:** Cells of non-differentiated cell mass in a hydrated larva. **C:** Fat body cells from a dried larva. **D**: Whole fat body cell from a hydrated larva. In the dry state, chromatin in the nuclei of both cell types showed clear segregation patterns. The chromatin of the cells from hydrated larvae is osmiophilic and widely distributed. **n** – nuclei. Bar = 1 µm; white arrows indicate location of invaginations in the membranes of the nuclei (**A**), white arrowheads indicate cell membrane of fat body cells (**C**, **D**).

Two types of cells in dry larvae were used for analysis of the state of chromatin: one comprises small cells with large nuclei occupying more than 50% of the total cell area and which form compact clusters (Fig. 2A, B), and the other consists of large cells with dense cytoplasm and which are abundant in the body (Fig. 2C, D). The latter category represents fat body cells and the former might be non-differentiated cells of hematopoietic organs or imaginal pads. These cells in fat body of the larvae have been shown earlier to be capable of surviving complete desiccation even if dissected from the larvae, which makes this cell type a convenient model for studies of anhydrobiosis [37], [38].

The clustered, non-differentiated cells of dehydrated and hydrated larvae showed no apparent difference in the size ratio of the nucleus to the cytoplasm. However, entry into anhydrobiosis resulted in deformation of the nuclear shape, and the formation of multiple invaginations (Fig. 2A, arrows), most probably reflecting general shrinkage of the cells upon dehydration and replacement of water with trehalose and other protectants (Fig. 2A, B). In fat body cells, in contrast, the total area of cytoplasm in anhydrobiotic larvae greatly decreased in comparison to the hydrated condition (Fig. 2D), while the nuclear shape and size did not exhibit obvious changes (Fig. 2C, D).

In both cell types, the nuclei of hydrated cells contained osmiophilic and widely dispersed chromatin (n, Fig. 2B, D). However, the nucleoplasm, particularly the dispersed chromatin, exhibited clear segregation and condensation patterns in dry cells (Fig. 2A, C). In *Polypedilum* larvae, even after 14 months of dry preservation, no further differences related to the duration of anhydrobiosis in morphology of cells and organelles were observed. The larvae which have been left for at least 24 h following complete rehydration still exhibited the condensation of chromatin in its nuclei, similar to that of larvae in the dry state.

### Antioxidant activity during anhydrobiosis cycle in the larvae

**Figure 3:**
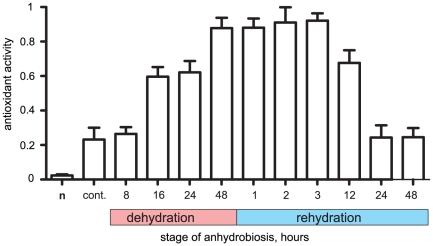
Relative antioxidant activity during dehydration/rehydration cycle, recalculated from ROS-scavenging ability of a *P. vanderplanki* larva during the course of dehydration and rehydration after anhydrobiosis. Error bars represent mean value ±95% CI for three replicates. **cont.** – control hydrated larvae. **n** –samples with crude from larvae not added.

We observed an initial elevation of total antioxidant activity (ROS-scavenging capacity) in the larvae subjected to dehydration for 16 h (D-16 h stage), which reached its maximum in completely dehydrated larvae at D-48 h (3–4 fold increase compared with unstressed wet larvae) (Fig. 3). Upon rehydration, high level of total antioxidant activity continued to be observable in the larvae for the first 12 h minimum (R-12 h), and was followed by the reduction to the same level as the non-stressed larvae at R-24 h.

### Identifying the presence of mature glutathione peroxidase protein in desiccated larvae

**Figure 4:**
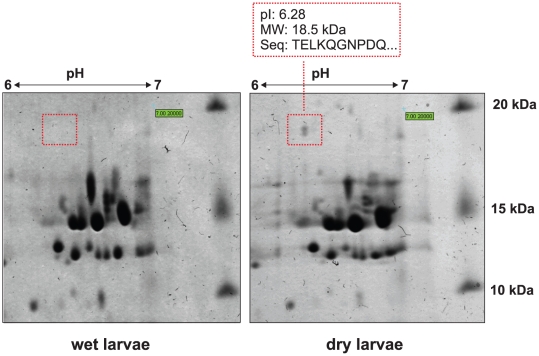
Accumulation of mature glutathione peroxidase in the desiccated larvae of the sleeping chironomid. In a fragment of 2D electrophoresis image of total proteins from **wet** (left image) and **dry** (right image) larvae the spot corresponding to the glutathione peroxidase is marked and estimated molecular weight, isoelectric point and read sequence of the protein are provided.

In order to isolate the essence of the antioxidant activity, a candidate spot was identified on 2D gel prepared using total protein crude from desiccated larvae (Fig. 4). The amino-acid sequence of the protein's N-terminus was found to be TELKQGNPDQ, which corresponds to the amino acids 30–39 of the protein product of glutathione peroxidase coding gene (HQ331115) and represents a mature enzyme (lacking signal peptide). No corresponding spot was detectable on the 2D electrophoresis gel prepared from hydrated active larvae thus we concluded that mature glutathione peroxidase accumulated in the anhydrobiotic larvae (Fig. 4, left panel).

### Gene expression in dehydrating, rehydrated, and irradiated larvae

#### Antioxidants

**Figure 5:**
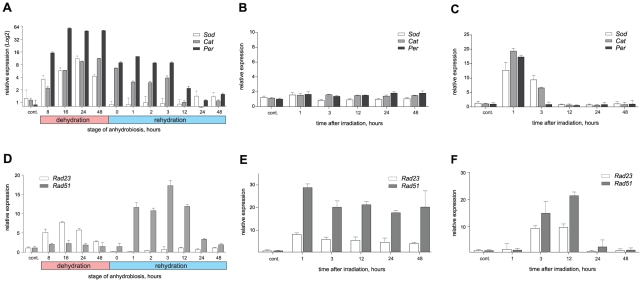
Relative mRNA expression profiles for selected genes encoding antioxidants (A, B and C) and DNA repair enzymes (D, E, F) in anhydrobiotic (A, D), heavy ion beam- (B, E) and gamma rays- (C, F) irradiated larvae. Values for the mRNA level of each gene were corrected for expression level of *EF1-alpha*, and the relative level of expression changes for each gene was calculated using that of control hydrated larvae as standard (value  = 1). Error bars represent mean value ±95% CI for three replicates. cont. – control hydrated larvae.

Three major components of the ROS elimination system (catalase (HM062769), Cu/Zn-superoxide dismutase (HM062770) and glutathione peroxidase (HQ331115)) are found to be abundant in the EST database prepared from larvae entering anhydrobiosis [32], [39]. Quantitative RT-PCR showed a high level of expression for all of these genes in the drying larvae by the D-8h stage, reaching a peak of an 8–10 fold increase of mRNA level at the D-24h and D-48h stages (Fig. 5A). During rehydration, there was no additional increase in the expression of these genes, but differences in their expression profiles were apparent: for the SOD encoding gene (*Sod*), mRNA levels immediately fell to control levels seen in hydrated larvae and maintained these levels throughout all stages of rehydration (R-1h to R-48h), while the expression of both catalase (*Cat*) and peroxidase (*Per*) encoding genes returned to the level of control hydrated larvae only at stage R-12h (Fig. 5A). The two types of radiation (^4^He ions and gamma-rays) resulted in different expression profiles of antioxidant-encoding genes. While no significant changes in expression of any of the three genes were observed in ^4^He-irradiated larvae (Fig. 5B), gamma-ray irradiation resulted in an increased expression of all three genes within the first few hours after irradiation before control levels were resumed (Fig. 5C).

#### DNA repair enzymes

We have analyzed the expression of two genes involved in DNA damage recognition and repair: *Rad23* (HM062772), whose protein plays a central role in proteosomal degradation of misfolded proteins but is also involved in both DNA excision repair and different types of DNA damage recognition [40]; and *Rad51* (HM062773), whose protein participates in a common DNA damage response pathway associated with the activation of homologous recombination and double-strand break (DSB) repair [41].

An increase in *Rad23* expression is observed in both ^4^He-irradiated and gamma-ray irradiated larvae (Fig. 5E, F). Heavy-ion irradiation resulted in an 8-fold increase of the *Rad23* mRNA expression in the larvae within an hour of irradiation, and the increased level of expression was maintained for at least 48 h (Fig. 5E). A transient up-regulation of *Rad23* was detectable in the larvae 3 h after gamma-ray irradiation, with mRNA levels decreasing to the control level during the next 24 h (Fig. 5F). Significant up-regulation of the *Rad23* gene was also found in dehydrating larvae beginning at the D-8h stage, and reaching a maximum value of 7-8-fold up-regulation at D-16h and -24h. Little or no expression of the gene was detected immediately after rehydration, but within a few hours (R-3h) the level of mRNA returned to the average control value (Fig. 5D).

Expression of the *Rad51* gene was up-regulated by more than 25-fold in the larvae within 1 h following heavy-ion irradiation and was maintained at a high level for at least 48 h (Fig. 5E). Similarly, an increase in *Rad51* mRNA level was induced by gamma-rays by 3 h after irradiation and remained high (more than 20-fold higher compared to control hydrated larvae) until at least the 12 h stage before returning to the control levels (Fig. 5F).

*Rad51* gene expression showed a significant increase during anhydrobiosis, but unlike *Rad23*, the increase in *Rad51* gene expression did not begin until rehydration. Maximal expression of *Rad51* was observed in the larvae after 3 h of rehydration following which expression gradually decreases and finally returns to a level comparable to control wet larvae at the R-48h stage (Fig. 5D).

## Discussion

Anhydrobiotic chironomid larvae can withstand an exposure to various external stresses, including high dose radiation [2], [7], [36], [42]. The molecular mechanisms which allow the chironomid larvae to survive an almost complete dehydration share some common features with those of other anhydrobionts. These are: (i) the replacement of structural water with compatible solutes such as disaccharides; (ii) the formation of stable glasses from highly hydrophilic proteins which prevent the biomolecules from irreversible aggregation [1], [5], [12], [21], [43]. However, water replacement and vitrification alone do not fully explain the cross-tolerance to different types of ionizing radiation. In the present study, we demonstrated that *P. vanderplanki* also has a remarkable resilience against DNA breaks caused by desiccation and two types of radiation.

We found that despite the presence of trehalose and other protectants associated with anhydrobiosis [32], [34], there were severe damages to nuclear DNA in the cells of anhydrobiotic larvae (Fig. 2). Simultaneously, antioxidant activity increased upon dehydration (Fig. 3), which is probably attributed to the elevation of ROS levels in the larvae. As suggested for other anhydrobionts by several authors [3], [44], the ROS generated during dehydration of cells may be the major cause of DNA fragmentation, and this is also likely to be true for *P. vanderplanki*. Slow dehydration provides an optimal condition to enter anhydrobiosis successfully [33], thus, in this case larvae are subjected to prolonged periods of “intermediate” water concentrations. However, we suppose that all metabolic processes, including respiration, nucleic acid synthesis and accumulation of protectants need to take place continuously until the intracellular matrix vitrifies [45], [46]. This state of water deficit is dangerous for the cells as it is associated with over-production of ROS [3], [17], [44].

An initial increase in the expression of genes involved in the oxidative stress response was observed in the desiccated larvae, followed by the elevation of total oxidants activity and the accumulation of mature antioxidants. These changes are likely to be due to an increase in ROS concentration triggered by the onset of desiccation (Fig. 3; Fig. 5A; Fig. 4). The antioxidants-coding genes were induced by low-LET (gamma-rays) radiation, which causes excessive ROS production, but not by high-LET radiation, which mainly exerts direct effects on biomolecules (Fig. 5 B, C). A gradual decrease of both the expression of these genes and the antioxidant activity in rehydrated larvae (Fig. 5A) would suggest that the consequences of ROS activity are neutralized, at least at the early stage of rehydration by the antioxidants synthesized before entering the dry state. This stays in agreement with the results from the analysis of EST databases prepared from anhydrobiotic larvae, which showed that the elevation in the expression of other antioxidants and heat shock protein-coding genes is tightly linked with anhydrobiosis in the larva [35], [39]. This accumulation of antioxidants, which maintains its activity even in the dry larvae might be one of the key factors ensuring the survival of *P. vanderplanki* in dry state, as it does in anhydrobiosis-capable cyanobacteria, plant seeds, resurrection plant tissues and nematodes [47], [48].

Nevertheless, the changes in chromatin ultrastructure (Fig. 2) and the occurrence of DNA breaks in the dried larvae (Fig. 1) suggest that, despite the activation of ROS-elimination systems (Fig. 3), the oxidative stress due to desiccation is not completely neutralized. Similar patterns of nuclei were observed in the fat body cells of the gall fly *Eufrosta* after high pressure freezing [49], and segregation of chromatin was taken to indicate DNA damage [50], [51], [52]. Furthermore, the presence of DNA breaks, and not that of ROS, is likely to be responsible for the induction of genes (*Rad23* and *Rad51*) directly involved in different types of DNA repair, as both genes were up-regulated by desiccation and both high- and low-LET radiation (Fig. 4D, E, F).

Although the process leading to the general recovery of nuclear DNA integrity in rehydrated larvae is still unclear, there are at least two possibilities: (i) fragmented DNA is restored by DNA repair systems; or (ii) damaged cells are eliminated by apoptosis while the remaining intact cells proliferate. The latter hypothesis seems less plausible, since we found continuous and gradual decrease in “comet tails” of damaged cells, suggesting that DNA reparation is taking place in either the rehydrated or the irradiated larvae (Fig. 2). The occurrence of rapid DNA repair that has been suggested by many authors to be a specific feature of anhydrobiotic organisms [1], [8], [10], [22], [29], [53] was not observed in the cells we studied. Instead, it took more than 48 h to complete DNA recovery in the larvae reviving after anhydrobiosis and even longer in larvae irradiated with ^4^He ions (Fig. 1). Typically, the repair of DSB in living cells takes less than 24 h and, in many cases, excess DNA damage in higher eukaryotes, including insects, triggers necrotic or apoptotic processes [54], [55], [56]. We still do not know how the larvae prevent cells with damaged DNA from committing apoptosis over such an extended period of time. Further cytological and biochemical studies must be carried out to resolve this issue since some observations suggest that there might be a specific regulation of apoptosis in anhydrobionts [57], [58], [59].

Recent studies have focused on survival rates after anhydrobiosis and showed that not all larvae are able to revive from the dry state; several physiological factors, including the rate of dehydration, determine the viability of the anhydrobiotic larvae [33]. Clearly water replacement and vitrification are indispensable for successful induction of anhydrobiosis [5], [34]. Nevertheless, our present data suggest that such protective mechanisms are insufficient for the maintenance of structural integrity of DNA in dry cells, and DNA repair after rehydration is another key for successful anhydrobiosis. Concerning this point, genetic adaptations to anhydrobiosis in the sleeping chironomid show some functional analogies with those of the radiotolerant bacteria *Deinococcus radiodurans* [23], [60], in which both desiccation and irradiation cause severe DNA damage, followed by prolonged DNA recovery period associated with delay in cell cycle (while doubling time under normal conditions is 1.5–3 h) [23], [60]. At the same time there are clear differences in these two phenomena, i.e., DNA reparation machinery and oxidative stress-response are different in eukaryotes and prokaryotes, genome organization in insects is much more complex and there is cell and tissue specification [61], [62], [63], [64]. In addition, recent studies have suggested that DNA breaks take place in other anhydrobionts such as bdelloid rotifers [30], [65]. Therefore, this convergent characteristics, as well as molecular protection by glasses, must be taken into account for future development of biotechnology, i.e., dried cell preservation.

The anhydrobiotic chironomid larvae presumably experience nuclear DNA fragmentation with each cycle of desiccation and rehydration, and must have overcome this threat efficiently to survive the drought season. It is likely that an initial increase in the expression of genes coding for antioxidants and DNA repair enzymes as well as the increase in antioxidant activity are rather typical reactions of common insects to desiccation stress [66], [67], [68], [69]. During the course of evolution, *P. vanderplanki* might have intensified this response, concomitantly with the acquisition of an ability to preserve the viability of cells beyond the dehydration threshold at which other insects would die. This anhydrobiosis-related evolution of augmented antioxidant protective mechanisms and DNA repair machinery is also most likely responsible for the remarkable cross-resistance of *P. vanderplanki* larvae in both dry and hydrated forms to the different types of ionizing radiation.

## Materials and Methods

### Insect rearing

*P. vanderplanki* were reared on a 1% agar diet containing 2% commercial milk under controlled light (13 h light: 11 h dark) and temperature (27–28°C) conditions according to previous report [31]. Final instar larvae of approximately 1 mg wet body weight were used for all experiments. The procedure of desiccation to induce anhydrobiosis has been described [70]. Briefly, the larvae were placed on filter paper with 0.44 ml of distilled water in a glass Petri dish (diameter 65 mm, height 20 mm), which was set in a desiccator (20×20×20 cm) with 1 kg of silica gel. For rehydration, dry larvae were placed in dishes with 27–28°C distilled water. Larvae for RNA expression analysis and antioxidants activity assay were sampled according to the time (in hours) passed from the beginning of desiccation (D) and rehydration (R), correspondingly.

### Irradiation

For gamma-ray irradiation, approximately 100 hydrated larvae were placed in a plastic vial (Sumilon MS-4503, Sumitomo Bakelite Co., Tokyo, Japan) with 1 ml water. The samples were irradiated with 70 Gy of gamma-rays from a ^60^Co source at 60 Gy/min [16]; 70 Gy is the half-inhibition gamma-ray dose for adult emergence in hydrated larvae [16].

For heavy-ion irradiation, hydrated larvae were placed on the bottom of a plastic Petri dish (diameter 50 mm, height 10 mm). The dish was covered with polyimide film and sealed with Parafilm (Alcan Packaging, Chicago, IL) to avoid drying. The samples were exposed to 70 Gy of a 50 MeV ^4^He (LET_∞_ = 16.2 keV/µm) ion beam delivered from the azimuthally varying-field (AVF) cyclotron at the Takasaki Ion accelerators for Advanced Radiation Application (TIARA) facility of the Japan Atomic Energy Agency (JAEA) [16], [36].

Control samples were sham irradiated and manipulated in parallel with the test samples. Both irradiated and non-irradiated larvae were supplied with distilled water.

### Source of clones

All clones of target genes used in this study were obtained by analysis of the Pv-EST database [32], [39]. The full-length cDNAs were subcloned into pCR4Blunt-TOPO vector (Invitrogen, Carlsbad, CA) and the resulting plasmids were used as templates for the calibration controls of real-time PCR reactions. DNA sequences were analyzed with Vector NTI 10.3 software (Invitrogen).

### Quantitative real-time PCR

Total RNA from hydrated, dehydrating, rehydrated, and irradiated larvae was extracted using Trizol (Invitrogen) and the RNeasy Mini Kit (Qiagen, Hilden, Germany), and reverse transcribed using Ready-To-Go™ T-Prime First-Strand Kit (GE Healthcare Bio-Sciences, Piscataway, NJ). The RNA samples from dehydrating and rehydrating larvae were named “D” and “R”, respectively, and numbers correspond to the hours of treatment. Real-time PCR was performed using a LightCycler ® 2.0 Real-Time PCR apparatus (Roche Diagnostics, Basel, Switzerland) with SYBR® Green PCR Master Mix (TaKaRa, Ohtsu, Japan).

Amplifications were performed using 1× SYBR Green PCR mix (TaKaRa) and 10 pmol of each primer. *P. vanderplanki EF1-alpha* cDNA served as an internal standard for data normalization and quantification. The expression of each gene was tested in triplicate in each of three biologically independent experiments. The cycling conditions were: 15 min activation at 95°C, 45 cycles of 10 s at 95°C, 20 s at 60°C, 25 s at 72°C. Melting curves from 60°C to 99°C, rising by 1°C at each step, and pausing 5 s after each step, and the accompanying software were used for qPCR data normalization and quantification. The genes, GenBank accession numbers, amplicon sizes and primers are shown in Table S1.

### Antioxidant activity (ROS-scavenging) assay

The antioxidant activity was investigated in the larvae during dehydration or rehydration, using an antioxidant activity assay kit (AB-2970 CLETA-S; Atto, Tokyo, Japan). Briefly, hypoxanthine-xanthine oxidase systems were used as the source of ROS. Chemiluminescence generation by the reaction between superoxide generator and a luminous substance, MPEC, and its decay in the presence of the crude from a homogenized single larva with assay buffer was measured. Relative antioxidant-scavenging capacity in a single larva was calculated according to manufacturer's manual.

### 2D electrophoresis, image analysis, and protein sequencing

Active (wet) and desiccated (dry) larvae were homogenized in T-PER lysis buffer (Pierce Biotechnology, Rockford, IL) with Complete protease inhibitor cocktail (Roche, Basel, Switzerland). Obtained crude protein samples (100 µg) were cleaned by 2-D Clean Up kit (GE Healthcare Bioscience), and applied to 11 cm IPG strips (pH 4–7, Bio-Rad, Hercules, CA) for passive overnight rehydration according to the manufacturer's instructions. The IPG strips were then subjected to isoelectric focusing using a PROTEAN IEF Cell (Bio-Rad). Focusing was performed for 38,000 V-hour. After isoelectric focusing, the IPG strips were equilibrated for 15 min in equilibration buffer I [6 M urea, 2% (w/v) SDS, 0.05 M Tris-HCl (pH 8.8), 20% (v/v) glycerol and 2% (w/v) dithiothreitol (DTT)] followed by 15 min in buffer II (same as buffer I but containing 2.5% iodoacetamide instead of DTT). For the second dimension, IPG strips were placed across a 17% acrylamide Gel for PROTEAN II D xi cell (Bio-Rad), then overlayed with agarose. Electrophoresis was run with a constant voltage, 140V, for 2 h in Tris-glycine buffer (25 mM Tris, 192 mM glycine, 0.1c SDS. pH 8.3). Gels were stained with Coomassie brilliant blue (CBB) G-250 solution for 30 min and washed in water and further distained by acetic acid-methanol solution two times, 30 min each. To obtain image files, stained gels were scanned with a high-resolution scanner (GT-X800, Epson, Tokyo, Japan). Protein spots were matched automatically by 2D Platinum© (GE Healthcare). Spot intensities were normalized to make the total density in each gel image equal, and analysis was performed using quantitative and qualitative modes. A spot was detected when its intensity was X?-fold or more above the background. The gels were blotted to PVDF membrane and stained with CBB R-250 and after destaining, the selected protein bands were cut out and used directly for sequencing by the Edman degradation method using a HP 241 Protein Sequencer according to the manufacturer's instructions.

### Comet Assay

Alkaline electrophoresis was performed using the CometAssay™ Kit (Trevigen, Gaithersburg, MD). Larvae were dissected and the fat body was extracted. Fat body cells were mixed with 95 µl of 1% low melting point agarose and spread on two slides previously coated with 1.5% normal agarose. After solidification by cooling, the slides were immersed in fresh lysis solution plus 10% DMSO for at least 45 min. The slides were incubated in alkaline buffer solution (300 mM NaOH and 1 mM EDTA, pH 12.6) for 25 min. The cells were subjected to electrophoresis for 25 min at 300 mA and 25 V, and then neutralized with 400 mM Tris-Cl, pH 7.5, in three successive washes of 5 min each. The DNA was then stained with ethidium bromide (2 µg/ml). Images of 100 randomly selected cells (from each of three replicate slides) were analyzed from each individual. Occasional dead cells, overlapping cells and cells on the edge of gels were avoided. Percentages of DNA in a comet “head” (intact DNA) and comet “tail” (damaged fragmented DNA) were determined by CometScore PC software (TriTek Corp, Sumerduck, VA).

### Transmission electron microscopy (TEM)

Dried and hydrated larvae were fixed in 2.5% glutaraldehyde in 50 mM phosphate buffer, pH 7.4 for 2 h at 4°C. The tissues were post-fixed in 2% osmium tetroxide in the same buffer for 1 h at 4°C. Dehydration of the tissues was conducted using an ethanol series of increasing concentration. Subsequently, the tissue pieces were embedded in a mixture of epoxy resins and were allowed to polymerize in a thermostat. The sections were contrasted by Na-uranyl acetate and Pb-citrate and observed with a JEM 100CX transmission electron microscope using the manufacturer's instructions. All cells and tissues of the larvae were identified according to their ultrastructure [71].

### Statistical analysis

Results of gene expression and the level of DNA damage are reported as means ±95% CI (confidence index, with *P*<0.05). The statistical evaluation was performed using a two-tailed Student t-test (Prism version 5, GraphPad Software, San Diego, CA).

## Supporting Information

Table S1 Primer pairs used for quantitative real-time PCR in this study.
(0.04 MB DOC)

## References

[1] Watanabe M (2006). Anhydrobiosis in invertebrates.. Applied Entomology and Zoology.

[2] Crowe LM, Crowe JH (1992). Anhydrobiosis: a strategy for survival.. Adv Space Res.

[3] Franca MB, Panek AD, Eleutherio EC (2007). Oxidative stress and its effects during dehydration.. Comp Biochem Physiol A Mol Integr Physiol.

[4] Simonin H, Beney L, Gervais P (2007). Sequence of occurring damages in yeast plasma membrane during dehydration and rehydration: mechanisms of cell death.. Biochim Biophys Acta.

[5] Hengherr S, Heyer AG, Kohler HR, Schill RO (2008). Trehalose and anhydrobiosis in tardigrades—evidence for divergence in responses to dehydration.. FEBS J.

[6] Clegg JS (2007). Protein stability in Artemia embryos during prolonged anoxia.. Biological Bulletin.

[7] Watanabe M, Nakahara Y, Sakashita T, Kikawada T, Fujita A (2007). Physiological changes leading to anhydrobiosis improve radiation tolerance in Polypedilum vanderplanki larvae.. J Insect Physiol.

[8] Gladyshev E, Meselson M (2008). Extreme resistance of bdelloid rotifers to ionizing radiation.. Proc Natl Acad Sci U S A.

[9] Daly MJ, Gaidamakova EK, Matrosova VY, Vasilenko A, Zhai M (2007). Protein oxidation implicated as the primary determinant of bacterial radioresistance.. PLoS Biol.

[10] Alpert P (2006). Constraints of tolerance: why are desiccation-tolerant organisms so small or rare?. J Exp Biol.

[11] Jonsson KI, Rabbow E, Schill RO, Harms-Ringdahl M, Rettberg P (2008). Tardigrades survive exposure to space in low Earth orbit.. Curr Biol.

[12] Hengherr S, Worland MR, Reuner A, Brummer F, Schill RO (2009). High-temperature tolerance in anhydrobiotic tardigrades is limited by glass transition.. Physiol Biochem Zool.

[13] Slade D, Lindner AB, Paul G, Radman M (2009). Recombination and replication in DNA repair of heavily irradiated Deinococcus radiodurans.. Cell.

[14] Yoshinaga K, Yoshioka H, Kurosaki H, Hirasawa M, Uritani M (1997). Protection by trehalose of DNA from radiation damage.. Biosci Biotechnol Biochem.

[15] Crowe JH, Oliver AE, Tablin F (2002). Is there a single biochemical adaptation to anhydrobiosis?. Integrative and Comparative Biology.

[16] Watanabe M, Sakashita T, Fujita A, Kikawada T, Horikawa DD (2006). Biological effects of anhydrobiosis in an African chironomid, Polypedilum vanderplanki on radiation tolerance.. Int J Radiat Biol.

[17] Kranner I, Birti S (2005). A Modulating Role for Antioxidants in Desiccation Tolerance1.. Integrative and Comparative Biology.

[18] Jonsson KI, Harms-Ringdahl M, Torudd J (2005). Radiation tolerance in the eutardigrade Richtersius coronifer.. Int J Radiat Biol.

[19] Hashimoto H, Greenberg M, Brack A, Colangeli L, Horneck G (1998). A conceptual design for cosmo-biology experiments in Earth's Orbit.. Biol Sci Space.

[20] Horneck G, Brack A (1992). Study of the origin, evolution and distribution of life with emphasis on exobiology experiments in earth orbit.. Adv Space Biol Med.

[21] Lapinski J, Tunnacliffe A (2003). Anhydrobiosis without trehalose in bdelloid rotifers.. FEBS Lett.

[22] Neumann S, Reuner A, Brummer F, Schill RO (2009). DNA damage in storage cells of anhydrobiotic tardigrades.. Comp Biochem Physiol A Mol Integr Physiol.

[23] Cox MM, Keck JL, Battista JR (2010). Rising from the Ashes: DNA Repair in Deinococcus radiodurans.. PLoS Genet.

[24] Huang Z, Boubriak I, Osborne DJ, Dong M, Gutterman Y (2008). Possible role of pectin-containing mucilage and dew in repairing embryo DNA of seeds adapted to desert conditions.. Ann Bot.

[25] Osborne DJ, Dell′Aquila A, Elder RH (1984). DNA repair in plant cells..

[26] Boubriak, Grodzinsky DM, Polischuk VP, Naumenko VD, Gushcha NP (2008). Adaptation and impairment of DNA repair function in pollen of Betula verrucosa and seeds of Oenothera biennis from differently radionuclide-contaminated sites of Chernobyl.. Ann Bot.

[27] Barrett J, Butterworth PE (1985). DNA stability in the anabiotic fourth-stage juveniles of Ditylenchus dipsaci (Nematoda).. Annals of Applied Biology.

[28] Rebecchi L, Cesari M, Altiero T, Frigieri A, Guidetti R (2009). Survival and DNA degradation in anhydrobiotic tardigrades.. J Exp Biol.

[29] McLennan AG (2009). Ametabolic embryos of Artemia franciscana accumulate DNA damage during prolonged anoxia.. Journal of Experimental Biology.

[30] Gladyshev EA, Arkhipova IR (2010). Genome structure of bdelloid rotifers: shaped by asexuality or desiccation?. J Hered.

[31] Watanabe M, Kikawada T, Minagawa N, Yukuhiro F, Okuda T (2002). Mechanism allowing an insect to survive complete dehydration and extreme temperatures.. J Exp Biol.

[32] Kikawada T, Nakahara Y, Kanamori Y, Iwata K, Watanabe M (2006). Dehydration-induced expression of LEA proteins in an anhydrobiotic chironomid.. Biochem Biophys Res Commun.

[33] Nakahara Y, Watanabe M, Fujita A, Kanamori Y, Tanaka D (2008). Effects of dehydration rate on physiological responses and survival after rehydration in larvae of the anhydrobiotic chironomid.. J Insect Physiol.

[34] Sakurai M, Furuki T, Akao K, Tanaka D, Nakahara Y (2008). Vitrification is essential for anhydrobiosis in an African chironomid, Polypedilum vanderplanki.. Proc Natl Acad Sci U S A.

[35] Gusev O, Cornette R, Kikawada T, Okuda T (2010). Expression of heat shock protein-coding genes associated with anhydrobiosis in an African chironomid Polypedilum vanderplanki.. Cell Stress Chaperones.

[36] Watanabe M, Sakashita T, Fujita A, Kikawada T, Nakahara Y (2006). Estimation of radiation tolerance to high LET heavy ions in an anhydrobiotic insect, Polypedilum vanderplanki.. Int J Radiat Biol.

[37] Watanabe M, Kikawada T, Fujita A, Okuda T (2005). Induction of anhydrobiosis in fat body tissue from an insect.. J Insect Physiol.

[38] Nakahara Y, Imanishi S, Mitsumasu K, Kanamori Y, Iwata KI (2009). Cells from an anhydrobiotic chironomid survive almost complete desiccation.. Cryobiology.

[39] Cornette R, Kanamori Y, Watanabe M, Nakahara Y, Gusev O (2010). Identification of anhydrobiosis-related genes from an expressed sequence tag database in the cryptobiotic midge Polypedilum vanderplanki (diptera; chironomidae).. J Biol Chem.

[40] Schauber C, Chen L, Tongaonkar P, Vega I, Lambertson D (1998). Rad23 links DNA repair to the ubiquitin/proteasome pathway.. Nature.

[41] Jia J, Tarabykina S, Hansen C, Berchtold M, Cygler M (2001). Structure of apoptosis-linked protein ALG-2: insights into Ca2+-induced changes in penta-EF-hand proteins.. Structure.

[42] Billi D (2009). Subcellular integrities in Chroococcidiopsis sp. CCMEE 029 survivors after prolonged desiccation revealed by molecular probes and genome stability assays.. Extremophiles.

[43] Crowe LM (2002). Lessons from nature: the role of sugars in anhydrobiosis.. Comparative Biochemistry and Physiology a-Molecular and Integrative Physiology.

[44] Blokhina O, Virolainen E, Fagerstedt KV (2003). Antioxidants, oxidative damage and oxygen deprivation stress: a review.. Annals of Botany.

[45] Kikawada T, Saito A, Kanamori Y, Nakahara Y, Iwata K (2007). Trehalose transporter 1, a facilitated and high-capacity trehalose transporter, allows exogenous trehalose uptake into cells.. Proc Natl Acad Sci U S A.

[46] Kikawada T, Saito A, Kanamori Y, Fujita M, Snigorska K (2008). Dehydration-inducible changes in expression of two aquaporins in the sleeping chironomid, Polypedilum vanderplanki.. Biochim Biophys Acta.

[47] Jenks MA, Wood AJ (2007). Plant desiccation tolerance..

[48] Reardon W, Chakrabortee S, Pereira TC, Tyson T, Banton MC (2010). Expression profiling and cross-species RNA interference (RNAi) of desiccation-induced transcripts in the anhydrobiotic nematode Aphelenchus avenae.. Bmc Molecular Biology.

[49] Morason RT, Allenspach AL, Lee RE (1994). Comparative ultrastructure of fat body cells of freeze-susceptible and freeze-tolerant Eurosta solidaginis larvae after chemical fixation and high pressure freezing.. Journal of Insect Physiology.

[50] Cleaver JE (1982). Normal reconstruction of DNA supercoiling and chromatin structure in cockayne syndrome cells during repair of damage from ultraviolet light.. Am J Hum Genet.

[51] Cuiffo BP, Fox HB, Babior BM (1985). Chromatin structure during bleomycin-induced DNA damage and repair.. J Free Radic Biol Med.

[52] Dinant C, Houtsmuller AB, Vermeulen W (2008). Chromatin structure and DNA damage repair.. Epigenetics Chromatin.

[53] Horikawa DD, Sakashita T, Katagiri C, Watanabe M, Kikawada T (2006). Radiation tolerance in the tardigrade Milnesium tardigradum.. Int J Radiat Biol.

[54] Cashio P, Lee TV, Bergmann A (2005). Genetic control of programmed cell death in Drosophila melanogaster.. Semin Cell Dev Biol.

[55] Kornbluth S, White K (2005). Apoptosis in Drosophila: neither fish nor fowl (nor man, nor worm).. Journal of Cell Science.

[56] Zhou L, Steller H (2003). Distinct pathways mediate UV-induced apoptosis in Drosophila embryos.. Developmental Cell.

[57] Menze MA, Hand SC (2007). Caspase activity during cell stasis: avoidance of apoptosis in an invertebrate extremophile, Artemia franciscana.. Am J Physiol Regul Integr Comp Physiol.

[58] Villenueve TS, Ma XC, Sun Y, Oulton MM, Oliver AE (2006). Inhibition of apoptosis by p26: implications for small heat shock protein function during Artemia development.. Cell Stress & Chaperones.

[59] Schill RO, Mali B, Dandekar T, Schnolzer M, Reuter D (2009). Molecular mechanisms of tolerance in tardigrades: New perspectives for preservation and stabilization of biological material.. Biotechnology Advances.

[60] Cox MM, Battista JR (2005). Deinococcus radiodurans - the consummate survivor.. Nat Rev Microbiol.

[61] Cromie GA, Connelly JC, Leach DRF (2001). Recombination at double-strand breaks and DNA ends: Conserved mechanisms from phage to humans.. Molecular Cell.

[62] Nickoloff JA, Hoekstra MF (1998). DNA damage and repair..

[63] Pitcher RS, Wilson TE, Doherty AJ (2005). New insights into NHEJ repair processes in prokaryotes.. Cell Cycle.

[64] Lushchak VI (2001). Oxidative stress and mechanisms of protection against it in bacteria.. Biochemistry-Moscow.

[65] Gladyshev EA, Meselson M, Arkhipova IR (2008). Massive horizontal gene transfer in bdelloid rotifers.. Science.

[66] Benoit JB, Del Grosso NA, Yoder JA, Denlinger DL (2007). Resistance to dehydration between bouts of blood feeding in the bed bug, Cimex lectularius, is enhanced by water conservation, aggregation, and quiescence.. American Journal of Tropical Medicine and Hygiene.

[67] Benoit JB, Lopez-Martinez G, Elnitsky MA, Lee RE, Denlinger DL (2009). Dehydration-induced cross tolerance of Belgica antarctica larvae to cold and heat is facilitated by trehalose accumulation.. Comparative Biochemistry and Physiology a-Molecular & Integrative Physiology.

[68] Benoit JB, Lopez-Martinez G, Elnitsky MA, Lee RE, Denlinger DL (2007). Moist habitats are essential for adults of the Antarctic midge, Belgica antarctica (Diptera: Chironomidae), to avoid dehydration.. European Journal of Entomology.

[69] Michaud MR, Benoit JB, Lopez-Martinez G, Elnitsky MA, Lee RE (2008). Metabolomics reveals unique and shared metabolic changes in response to heat shock, freezing and desiccation in the Antarctic midge, Belgica antarctica.. Journal of Insect Physiology.

[70] Watanabe M, Kikawada T, Okuda T (2003). Increase of internal ion concentration triggers trehalose synthesis associated with cryptobiosis in larvae of Polypedilum vanderplanki.. J Exp Biol.

[71] King RC, Akai H (1982). Insect ultrastructure..

