# Supplementary material for: Anhydrobiosis-Associated Nuclear DNA Damage and Repair in the Sleeping Chironomid: Linkage with Radioresistance
Source: PLoS One. 2010 Nov 16;5(11):e14008. doi: 10.1371/journal.pone.0014008 (PMC2982815; doi:10.1371/journal.pone.0014008)
Supplement: Table S1 — Primer pairs used for quantitative real-time PCR in this study. (0.04 MB DOC) [file pone.0014008.s001.doc]

Supplementary Table 1.

Primer pairs used for quantitative real-time PCR in the present study.

| Gene name / (GenBank accession number) | Primer name | Sequence | Product size (bp) |
| --- | --- | --- | --- |
| Cu/Zn-superoxide dismutase (HM062770) | SOD1-F | 5’-ttcgtcacgttggtgatt-3’ | 196 |
|  | SOD1-R | 5’-cgtccaccagcatttcc-3’ |  |
| Glutathione peroxidase  (HQ331115) | GPx-F | 5’-gggatgtataaaggctcatacg-3’ | 167 |
|  | GPx-R | 5’-cgccattttgtcaacaataaatttag-3’ |  |
| Catalase (HM062769) | Cat-F | 5’-gaaagctgaggaattggca-3’ | 153 |
|  | Cat-R | 5’-acatcaaatggattccatttgtat-3’ |  |
| Rad51 (HM062773) | Rad51-F | 5’-atttcaattacaactggatcacg -3’ | 232 |
|  | Rad51-R | 5’- gttcggctatagatgacaatctttt-3’ |  |
| Rad23 (HM062772) | Rad23-F | 5’-caagccagtcacaaattgc-3’ | 157 |
|  | Rad23-R | 5’-tattctactggctcgctcgg-3’ |  |
| EF1-alpha (AB490338.1) | EF1a-F | 5’-aactgacaaaccattgcg-3’ | 195 |
|  | EF1a-R | 5’-tcacctggtacagcttct-3’ |  |
